# Lysine Deprivation Induces AKT-AADAT Signaling and Overcomes EGFR-TKIs Resistance in *EGFR*-Mutant Non-Small Cell Lung Cancer Cells

**DOI:** 10.3390/cancers13020272

**Published:** 2021-01-13

**Authors:** Chia-Chi Hsu, Albert Ying-Po Yang, Jui-Yi Chen, Hsin-Hui Tsai, Shu-Heng Lin, Pei-Chen Tai, Ming-Hung Huang, Wei-Hsun Hsu, Anya Maan-Yuh Lin, James Chih-Hsin Yang

**Affiliations:** 1 Graduate Institute of Oncology, College of Medicine, National Taiwan University, Taipei 100, Taiwan; cchsu2@ntu.edu.tw (C.-C.H.); albertyang36@ntu.edu.tw (A.Y.-P.Y.); b07401011@ntu.edu.tw (J.-Y.C.); hsinhuitsai@ntu.edu.tw (H.-H.T.); shlin567@ntu.edu.tw (S.-H.L.); blairtai1994@ntu.edu.tw (P.-C.T.); mh122866@gmail.com (M.-H.H.); whhsu1977@ntu.edu.tw (W.-H.H.); 2 Cancer Biology Research Group, Center of Precision Medicine, National Taiwan University, Taipei 100, Taiwan; 3 Department of Oncology, National Taiwan University Hospital, Taipei 100, Taiwan; 4 National Taiwan University Cancer Center, Taipei 100, Taiwan; 5 School of Medicine, National Taiwan University, Taipei 100, Taiwan; 6 Faculty of Pharmacy, National Yang-Ming University, Taipei 112, Taiwan; 7 Department of Medical Research, Taipei Veterans General Hospital, Taipei 112, Taiwan

**Keywords:** *EGFR*-mutant NSCLC, lysine deprivation, AADAT, EGFR-TKI resistance, osimertinib

## Abstract

**Simple Summary:**

In the Asian population, 50–60% of non-small cell lung cancer (NSCLC) patients carry the epidermal growth factor receptor (*EGFR*) mutation. Although treatment with EGFR-tyrosine kinase inhibitors (EGFR-TKIs) is effective, resistance inevitably occurs. Moreover, previous studies showed that cancers harboring a specific mutation are sensitive to deficiency related to a particular amino acid. The identity of this amino acid is, however, unclear in the case of *EGFR*-mutant NSCLC. Our studies aim to identify the critical amino acid affected in *EGFR*-mutant NSCLC and develop a strategy against EGFR-TKI resistance. We determined that lysine is essential for the survival of *EGFR*-mutant NSCLC and EGFR-TKI-resistant sublines. In addition, we found that the presence of lysine reduction can lower the dosage of EGFR-TKI required for treatment in the case of *EGFR*-mutant NSCLC. Lastly, our findings provide a guiding principle showing that amino acid stress can enhance not only the therapeutic potential but also the quality of life for *EGFR*-mutant NSCLC patients.

**Abstract:**

Epidermal growth factor receptor (*EGFR*) mutations are the most common driver genes in non-small cell lung cancer (NSCLC), especially in the Asian population. Although EGFR-tyrosine kinase inhibitors (TKIs) are influential in the treatment of *EGFR*-mutant NSCLC patients, acquired resistance inevitably occurs. Therefore, there is an urgent need to develop strategies to overcome this resistance. In addition, cancer cells with particular mutations appear more vulnerable to deficiency related to the availability of specific amino acids. However, it is still unknown which amino acid is affected in the case of *EGFR*-mutant NSCLC. In the present study, we established a screening platform based on amino acid deprivation and found that *EGFR*-mutant NSCLC cells are sensitive to short-term lysine deprivation. Moreover, we found that expression of the gene for the lysine catabolism enzyme α-aminoadipate aminotransferase (*AADAT*) increased under lysine deprivation, revealing that *AADAT* can be regulated by EGFR–AKT signaling. Finally, we found that lysine reduction can not only enhance the cytostatic effect of single-agent osimertinib but also overcome the resistance of EGFR-TKIs in *EGFR*-mutant NSCLC cells. In summary, our findings suggest that the introduction of lysine stress might act as an advancement in *EGFR*-mutant NSCLC therapy and offer a strategy to overcome EGFR-TKI resistance.

## 1. Introduction

*EGFR* mutations are the most common driver genes and can be found in 50–60% of non-small cell lung cancer (NSCLC) patients in the Asian population, especially in women and non-smokers [1,2]. The ELREA in-frame deletion in exon 19 and L858R substitution in exon 21 are the most common (approximately 85%) among the activating EGFR mutations and are regarded as so-called classic mutations [3,4]. EGFR-tyrosine kinase inhibitors (EGFR-TKIs), including first-generation (gefitinib and erlotinib) and second-generation (afatinib) EGFR-TKIs, have been developed to target and effectively inhibit tumors harboring activating *EGFR* mutations [5,6]. Although first- and second-generation EGFR-TKIs benefit patients with NSCLC who carry activating *EGFR* mutations, acquired resistance always occurs within a median of 10–14 months under EGFR-TKI therapy [4,7,8]. The acquired T790M mutation in exon 20 of *EGFR* is the most common mechanism for resistance to first- and second-generation EGFR-TKIs (occurring in approximately half of EGFR-TKI resistance cases) [9]. The third-generation EGFR-TKI, osimertinib, was designed to target the acquired T790M mutation and the activating mutations of *EGFR*, while sparing wild type EGFR [10,11,12]. However, all patients eventually develop acquired resistance to osimertinib after a median of approximately 12 months of treatment. Therefore, it is urgent to find a strategy to target *EGFR*-mutant NSCLC and overcome drug resistance.

Besides intrinsic factors (genetic alterations in cancer cells), extrinsic factors, such as nutrient availability, oxygen concentration, and microenvironment, are also critical to tumor progression [13,14]. In addition, included are amino acids, which can be divided into essential amino acids (EAAs; His, Ile, Leu, Lys, Met, Phe, Thr, Trp, and Val), conditional EAAs (Arg, Cys, Gln, Gly, Pro, and Tyr), and non-essential amino acids (NEAAs; Ala, Asp, Asn, Glu, and Ser). These amino acids play a vital role in the physiological/metabolic functions of humans, and the abnormal metabolism of amino acids is related to many human diseases, including cancer [15,16,17,18]. Increasing evidence shows that a particular type of cancer or oncogenic mutation can lead cancer cells to rely on specific EAAs for survival, causing cancer cells to be sensitive to stresses related to specific EAA [19]. For example, melanoma, which activates RAS–BRAF–MEK1 signaling via oncogenic mutations, is very sensitive to leucine deprivation [20], and methionine depletion can inhibit cell growth and sensitize cancer cells to lexatumumab in triple-negative breast cancer (TNBC) [21]. *KRAS*G12D/*TP53*-depleted NSCLC incorporates free branched-chain amino acids (BCAAs; valine, leucine, and isoleucine) and uses BCAAs as a source of nitrogen, which is essential for tumor growth [22]. Further, lymphoblastic leukemia requires tryptophan and valine for survival [23,24]. Therefore, manipulation of specific amino acids might offer a strategy to treat particular types of cancer depending on their oncogenic mutations [19]. However, which amino acids are imperative in *EGFR*-mutant NSCLC remains unknown.

This study aimed to reveal the crucial amino acids in *EGFR*-mutant NSCLC cells that might serve in the development of therapies for treating *EGFR*-mutant NSCLCs. In this study, we determined that the *EGFR*-mutant and EGFR-TKI-resistant NSCLC cells are sensitive to lysine deprivation and that the cytostatic effects induced by these stresses are not observed in normal human lung fibroblasts. In addition, we found that a lysine catabolism enzyme, alpha-aminoadipate aminotransferase (AADAT), can be regulated by EGFR–AKT signaling. Moreover, we determined that the downregulation of AADAT by EGFR-TKIs can be observed in *EGFR*-mutant NSCLC, rather than EGFR-TKI-resistant NSCLC cells. Finally, we found that the presence of lysine reduction can enhance the cytostatic effect of osimertinib treatment in *EGFR*-mutant NSCLC cells. To conclude, our findings suggest that the induction of lysine stress might be a strategy to improve the therapy for *EGFR*-mutant NSCLCs and overcome resistance to EGFR-TKIs.

## 2. Results

### 2.1. The EGFR-Mutant NSCLC Cells Were Consistently Sensitive to Lysine Deprivation

**Figure 1:**
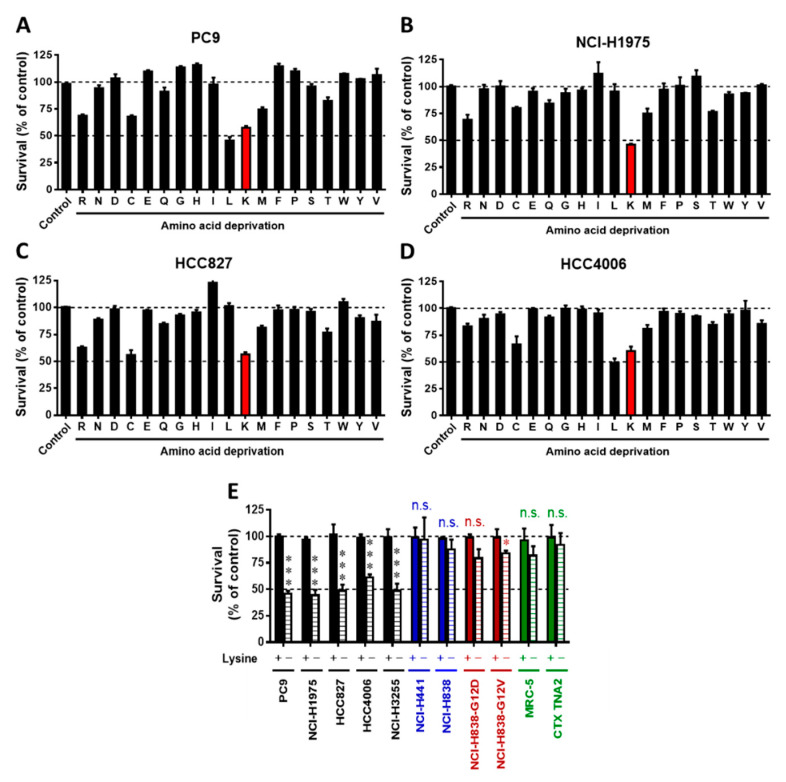
The epidermal growth factor receptor (*EGFR*)-mutant non-small cell lung cancer (NSCLC) cells were consistently sensitive to lysine deprivation. *EGFR-*mutant NSCLC cells ((**A**): PC9, (**B**): NCI-H1975, (**C**): HCC827, and (**D**): HCC4006) were deprived of each amino acid for 48 h, and their survival was analyzed using an SRB assay. (**E**) The response to lysine deprivation in *EGFR*-mutant NSCLC cells (black bar: PC9, NCI-H1975, HCC827, HCC4006, and NCI-H3255), *EGFR* wild-type NSCLC cells (blue bar: NCI-H441 and NCI-H838; dark red bar: *KRAS*-G12D and *KRAS*-G12V isogenic cells), and normal cells (green bar: normal human lung MRC-5 fibroblasts and normal rat astrocytes, CTX TNA2). The SRB analysis results shown are representative of three independent experiments. The data are presented as the mean ± SD. (***, *p* < 0.001; *, *p* < 0.05 compared with the control); n.s., non-significant; SRB, sulforhodamine B. The concentration of each amino acid used is described in Table A1 in Appendix A.

To determine which specific amino acid is crucial for *EGFR*-mutant NSCLC cell survival, we established a screening platform for amino acid stress by depriving individual amino acids (Figure 1). Cell survival was measured using this platform after 48 h of incubation of culture media containing different amino acid combinations. We found that PC9 was sensitive to arginine (R), cysteine (C), leucine (L), lysine (K), and methionine (M) (Figure 1A). NCI-H1975 was sensitive to the deprivation of arginine (R) and lysine (K) (Figure 1B). HCC827 was sensitive to arginine (R), cysteine (C), and lysine (K) (Figure 1C). HCC4006 was sensitive to the deprivation of cysteine (C), leucine (L), and lysine (K) (Figure 1D). These results indicate that the survival of *EGFR*-mutant NSCLC cells was differentially affected depending on the amino acid that was lacking. However, the survival of all *EGFR*-mutant NSCLC cells was consistently reduced by lysine deprivation (Figure 1E). By contrast, short-term lysine deprivation did not alter the survival of *EGFR* wild-type NSCLC cells, including NCI-H441 (*KRAS*-mutant), NCI-H838, and two isogenic cell lines (NCI-H838-*KRAS*G12V and NCI-H838-*KRAS*G12D) (Figure 1E; blue and brown bars) (Figure S1). At the same time, short-term lysine deprivation did not affect the survival of normal human lung fibroblasts (MRC-5) and normal rat astrocytes (CTX TNA2) (Figure 1; green bars). The results indicate that only *EGFR*-mutant NSCLC cells, but not *EGFR* wild-type or isogenic *KRAS*-mutant NSCLC cells, are sensitive to lysine deprivation. These results suggest that *EGFR*-mutant NSCLC cells are consistently sensitive to lysine deprivation.

**Figure 2:**
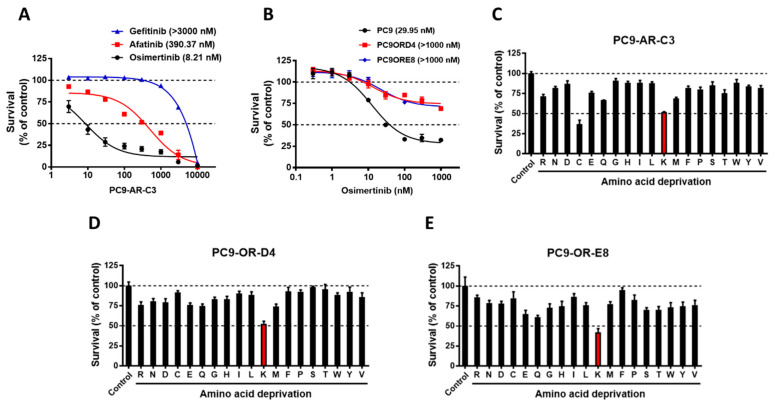
The EGFR-tyrosine kinase inhibitors (TKI)-resistant NSCLC cells were consistently sensitive to lysine deprivation. (**A**) The EGFR-TKI-resistant PC9 subline, PC9-AR-C3, was treated with various doses of gefitinib, afatinib, and osimertinib. Cell survival was analyzed using an SRB assay; (**B**) The parental PC9 cell lines and EGFR-TKI-resistant PC9 sublines, PC9-OR-D4 and PC9-OR-E8, were treated with several doses of osimertinib for 48 h. The survival of each cell line was analyzed using an SRB assay; (**C**) PC9-AR-C3, (**D**) PC9-OR-D4, and (**E**) PC9-OR-E8 cells were deprived of each amino acid for 48 h, and their survival was analyzed using an SRB assay (red bar: lysine deprivation). The results are representative data obtained from three independent experiments. OR, osimertinib-resistant cell line; AR, afatinib-resistant cell line; SRB, sulforhodamine B.

Those undergoing EGFR-TKI therapy reportedly develop acquired resistance after 10–14 months of treatment. The search for overcoming resistance to EGFR-TKIs in *EGFR*-mutant NSCLC has thus attracted increasing attention. Next, we developed an afatinib-resistant subline (PC9-AR-C3) and osimertinib-resistant sublines (PC9-OR-D4 and PC9-OR-E8). Sanger sequencing showed that PC9-AR-C3 harbored an acquired T790M mutation in exon 20 of *EGFR* (Figure S2), causing gefitinib and afatinib resistance but sensitivity to osimertinib (Figure 2A and Figure S3). In addition, osimertinib potently attenuated the survival of PC9 but not the osimertinib-resistant clones (PC9-OR-D4 and PC9-OR-E8) (Figure 2B). We further confirmed that the phosphorylation of AKT was maintained under afatinib treatment in PC9-AR-C3 when compared with the parental PC9 cells (Figure S3). Similarly, osimertinib treatment significantly reduced AKT phosphorylation in parental PC9 but not PC9-OR-D4 and PC9-OR-E8 cells (Figure S4). In the present study, the effect of lysine deprivation on the survival of EGFR-TKI-resistant PC9 sublines was investigated using the same screening platform whereby lines were deprived of specific amino acids, as shown in Figure 1. Similarly to the survival of *EGFR*-mutant NSCLC cells (Figure 1), lysine deprivation consistently reduced the survival of the three resistant cell lines (Figure 2C–E; red bars), and NCI-H1975 cells harboring exon-16-skipping human epidermal growth factor receptor 2 (HER2) (Figure S6) which, based on results from our recent study, is known to be an osimertinib-resistant cell line [25].

**Figure 3:**
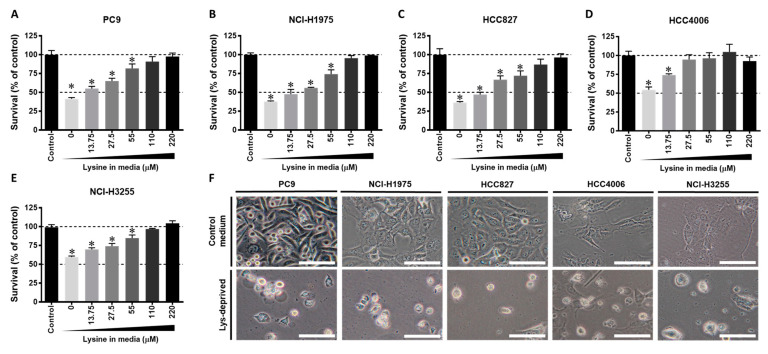
The effects of lysine reduction on *EGFR*-mutant NSCLC cells. (**A**) PC9, (**B**) NCI-H1975, (**C**) HCC827, (**D**) HCC4006, and (**E**) NCI-H3255 cells were incubated in culture media at a series of lysine concentrations for 48 h, and their survival was analyzed using an SRB assay. (**F**) *EGFR*-mutant NSCLC cells were incubated with a lysine-deprived media for 48 h, and representative images of the morphology were obtained using a light microscope (scale bar: 100 μm). The SRB analysis results shown are representative of three independent experiments. SRB, Sulforhodamine B. The data are presented as the mean ± SEM (*, *p* < 0.05 compared with the control).

Lysine is an essential amino acid that is not synthesized de novo and must be taken up by cells from the extracellular environment. Next, the dependence of lysine on the survival of *EGFR*-mutant NSCLC cells was evaluated. *EGFR*-mutant NSCLC cells (PC9, NCI-H1975, HCC827, HCC4006, and NCI-H3255) were incubated with or without lysine at concentrations ranging from 0 to 220 in RPMI 1640 medium). The results consistently demonstrated that the survival of all *EGFR*-mutant NSCLC cell lines was reduced under lysine deprivation (Figure 3A–E). In addition, we found that lysine deprivation resulted in cell-rounding morphology and a loss of adhesion in PC9, NCI-H1975, HCC827, HCC4006, and NCI-H3255 (Figure 3F). In conclusion, the results suggest that the survival of *EGFR*-mutant NSCLC cells is dependent on the abundance of lysine.

### 2.2. Lysine Deprivation Induces the Expression of α-Aminoadipate Aminotransferase in the Lysine Catabolism Pathway and Downstream EGFR Signaling in EGFR-Mutant NSCLC Cells

**Figure 4:**
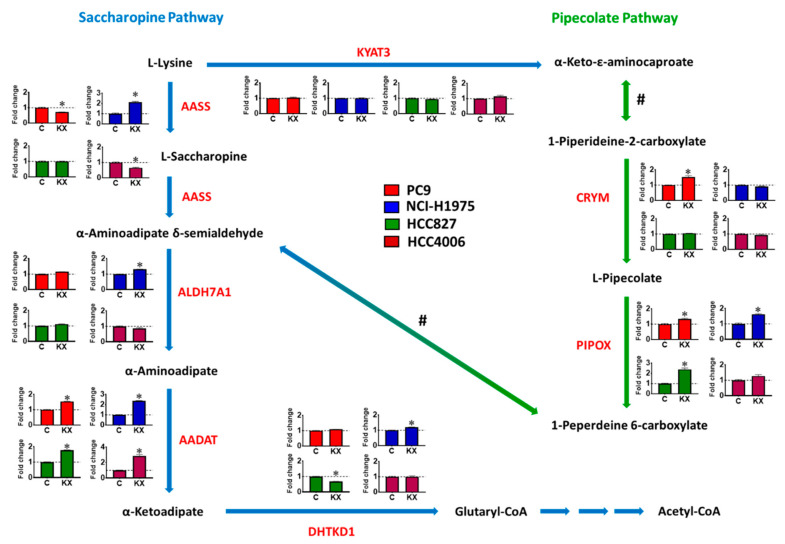
The effects of lysine deprivation on the expression of genes of the lysine catabolism pathway in *EGFR*-mutant NSCLC cells. The graph illustrates the mechanisms of the saccharopine pathway (blue line) and the pipecolate pathway (green line). Each *EGFR*-mutant NSCLC cell line (red bar: PC9, blue bar: NCI-H1975, green bar: HCC827, purple bar: HCC4006) was incubated with medium corresponding to either lysine-deprived (0 µM lysine) or control (220 µM lysine) conditions for 24 h. The expression of genes involved in lysine catabolism was then analyzed using qPCR. The relative fold change in expression levels for each gene in each lysine-deprived group was normalized according to the corresponding control. The result is representative of three independent experiments. C, normal RPMI; KX, lysine-deprived RPMI; *AADAT*, kynurenine/α-aminoadipate aminotransferase; *AASS* (L-lysine to L-saccharopine), α-aminoadipic semialdehyde synthase; *AASS* (L-saccharopine to α-aminoadipate δ-semialdehyde), saccharopine dehydrogenase; *ALDH7A1*, α-aminoadipic semialdehyde dehydrogenase; *CRYM*, ketamine reductase; *DHTKD1*, 2-oxadipate dehydrogenase; *KYAT3*, kynurenine aminotransferase 3; *PIPOX*, L-pipecolate oxidase; #, spontaneous and nonenzymatic. The data are presented as the mean ± SEM from three-independent experiments. (*, *p* < 0.05 compared with the normal RPMI group).

**Figure 5:**
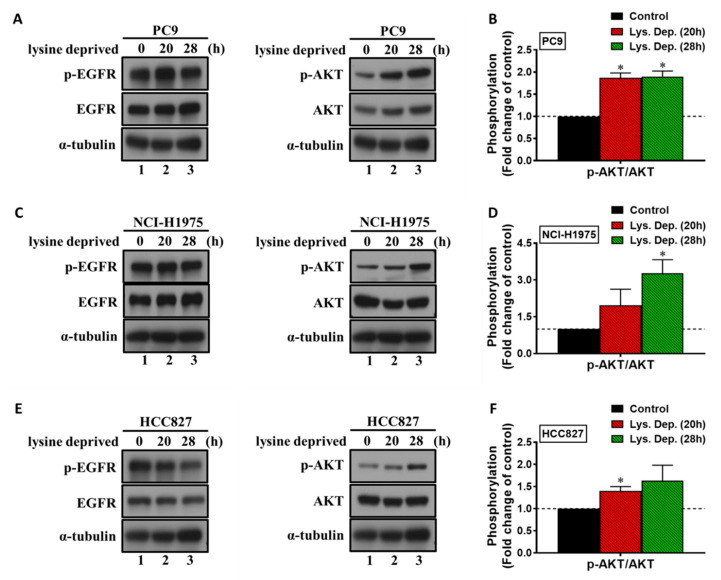
Lysine deprivation induces the phosphorylation of EGFR downstream targets. (**A**,**B**) PC9, (**C**,**D**) NCI-H1975, and (**E**,**F**) HCC827 cells were incubated with lysine-deprived RPMI for 20 and 28 h, and whole-cell extracts were used for Western blot analysis with antibodies against phosphor-EGFR (p-EGFR), EGFR, phosphor-AKT (p-AKT), AKT, and α-tubulin. Western blot analysis of each relative fold change was measured using the ImageJ software, normalized to each control group. The results shown are representative of three independent experiments. The data are presented as the mean ± SEM (*, *p* < 0.05 compared with the control group). Lys. Dep., lysine deprivation. Uncropped Western Blots can be found in Figures S7 and S8.

To investigate the effects of lysine deprivation on lysine catabolism pathways, we characterized the expression of genes encoding lysine catabolism enzymes. We found that lysine deprivation consistently induced only the expression of AADAT in all *EGFR*-mutant NSCLC cells and EGFR-TKI-resistant sublines (Figure 4 and Figures S3B and S4B,D). Next, we investigated the effects of lysine deprivation on EGFR-signaling in *EGFR*-mutant NSCLC cells. These results show that phosphor-AKT, a downstream target of EGFR signaling, was activated under lysine deprivation (Figure 5A,B for PC9; Figure 5C,D for NCI-H1975; and Figure 5E,F for HCC827). We also found that phosphor-AKT was activated under lysine deprivation in EGFR-TKI-resistant sublines (Figures S3C and S5C,E). These results suggest that lysine deprivation provokes both the expression of *AADAT* in the lysine catabolism pathway and the EGFR downstream target phosphor-AKT in *EGFR*-mutant NSCLC cells.

### 2.3. AADAT Is the Downstream Target of EGFR–AKT Signaling in EGFR-Mutant NSCLC Cells

**Figure 6:**
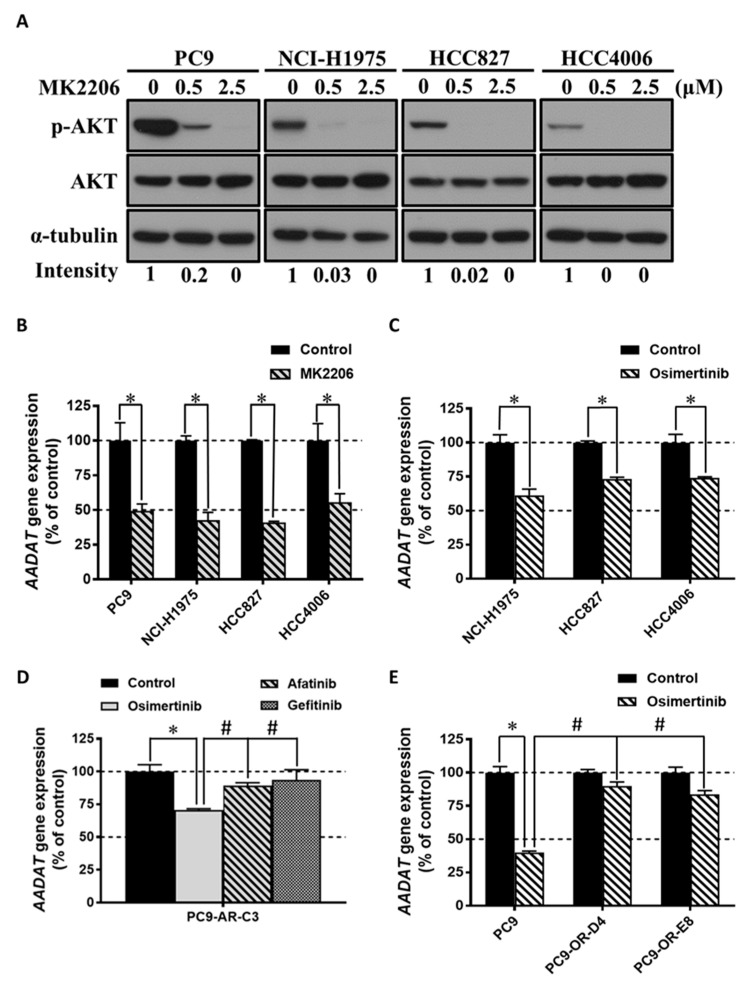
Inhibition of EGFR–AKT signaling decreased the expression of the AADAT gene in *EGFR*-mutant NSCLC cells. (**A**) PC9, NCI-H1975, HCC827, and HCC4006 were incubated with the RPMI 1640 medium along with 0.5 μM and 2.5 μM MK2206 for 3 h, and whole-cell extracts were used for the Western blot analysis with antibodies against phosphor-AKT (p-AKT), AKT, and α-tubulin. The Western blot analysis of p-AKT/AKT intensity was measured using the ImageJ software, normalized to each control group; (**B**) PC9, NCI-H1975, HCC827, and HCC4006 were incubated with an RPMI 1640 medium along with 1 μM MK2206; (**C**) NCI-H1975, HCC827, and HCC4006. Each of the *EGFR*-mutant NSCLC cells was incubated with an RPMI 1640 medium along with 50 nM osimertinib. The expression of the AADAT gene was analyzed using qPCR; (**D**) PC9-AR-C3; (**E**) PC9, PC9-OR-D4, and PC9-OR-E8. Each of the EGFR-TKI-resistant PC9 sublines and parental PC9 cell lines was incubated with an RPMI 1640 medium along with 50 nM osimertinib. The expression of the AADAT gene was analyzed using qPCR. The results are representative data obtained from two independent experiments. The data are presented as the mean ± SEM (*, *p* < 0.05 compared with the control group; #, *p* < 0.05 compared with the parental PC9 cells or osimertinib group). Uncropped Western Blots can be found in Figure S7.

*EGFR*-mutant NSCLC cells are known to rely on EGFR signaling for their survival, and we found that lysine deprivation can induce the expression of *AADAT*, as well as EGFR downstream signaling and the phosphorylation of AKT. Thus, we examined the relationship between *AADAT* expression and EGFR–AKT signaling in *EGFR*-mutant NSCLC cells. We used MK2206, which is an allosteric AKT inhibitor, to suppress the phosphorylation of AKT in four *EGFR*-mutant NSCLC cell lines (PC9, NCI-H1975, HCC827, and HCC4006) (Figure 6A). We found that the expression of *AADAT* decreased in response to MK2206 treatment in four *EGFR*-mutant NSCLC cells (Figure 6B). These results indicate that AKT can regulate the expression of *AADAT* in *EGFR*-mutant NSCLC cells. We next used osimertinib to suppress EGFR–AKT signaling in *EGFR*-mutant NSCLC cells and found that the incubation of osimertinib led to decreased expression of *AADAT* in NCI-H1975, HCC827, and HCC4006 (Figure 6C). To further confirm the regulation of *AADAT* by EGFR–AKT signaling in *EGFR*-mutant NSCLC cells, we treated an afatinib-resistant subline (PC9-AR-C3) with different generations of EGFR-TKIs, including gefitinib (first generation), afatinib (second generation), and osimertinib (third generation). We found that treatment with osimertinib, rather than gefitinib and afatinib, reduced *AADAT* expression in PC9-AR-C3 cells (Figure 6D), and the effects on the expression of *AADAT* under treatment with EGFR-TKI were consistent with the results of survival (Figure 2A). We also found that downregulation of *AADAT* expression by osimertinib treatment could only be observed in parental PC9 cells but not in the osimertinib-resistant clones (PC9-OR-D4 and PC9-OR-E8) (Figure 6E). These results demonstrate that *AADAT* is a downstream target of EGFR–AKT signaling in *EGFR*-mutant NSCLC cells.

### 2.4. Lysine Reduction not Only Reduced the Survival of EGFR-TKI-Resistant NSCLC Cells but Also Enhanced the Cytostatic Effect of the AKT Inhibitor and Osimertinib in EGFR-Mutant NSCLC Cells

**Figure 7:**
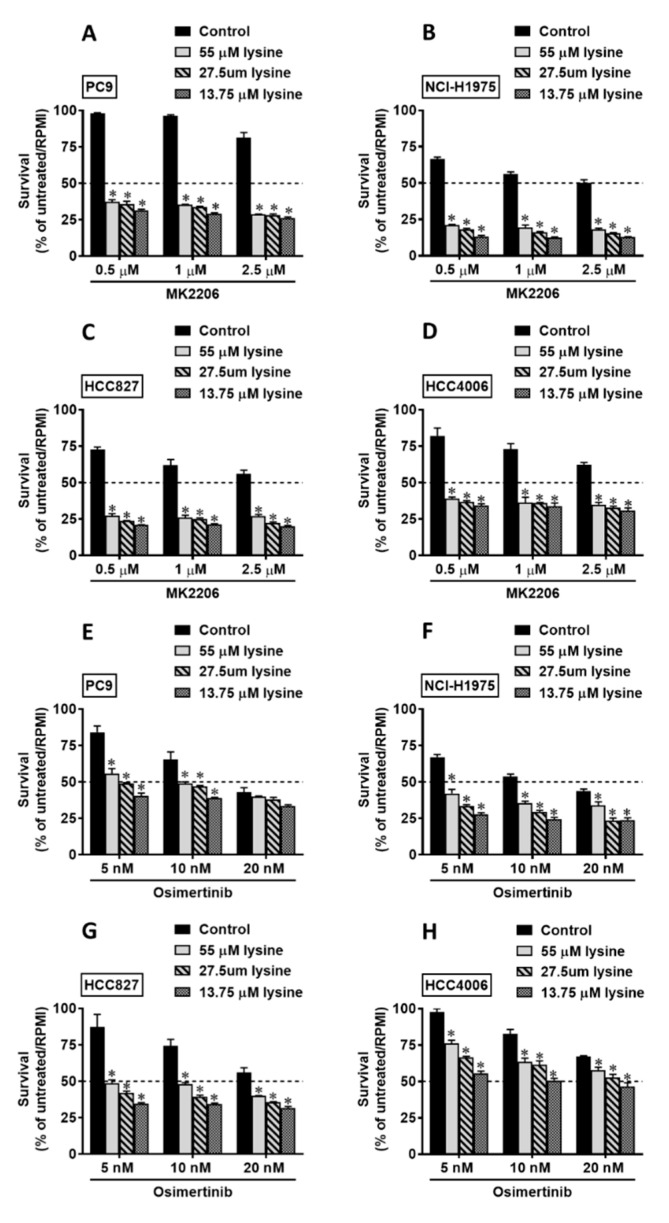
*EGFR*-mutant NSCLC cell survival strongly reduced under the combined treatment of MK2206 and osimertinib with lysine reduction. (**A**) PC9; (**B**) NCI-H1975; (**C**) HCC827; and (**D**) HCC4006 cells were incubated with lysine-deprived media containing 55, 27.5, and 13.75 µM lysine 0, 1, and 2.5 µM MK2206. (**E**) PC9; (**F**) NCI-H1975; (**G**) HCC827; and (**H**) HCC4006 cells were incubated with lysine-deprived media containing 55, 27.5, and 13.75 µM lysine along with 5, 10, and 20 nM osimertinib. The survival of each cell line was analyzed using an SRB assay. The results are representative data obtained from two independent experiments. The data are presented as the mean ± SEM (*, *p* < 0.05 compared with the control group); SRB, sulforhodamine B.

Because the phosphorylation of AKT was upregulated under lysine deprivation in the *EGFR*-mutant NSCLC cells (Figure 5), we next investigated the role of AKT under lysine stress in *EGFR*-mutant NSCLC cells. We treated each *EGFR*-mutant NSCLC cell line with the AKT inhibitor, MK2206, using different concentrations of lysine in the culture media (55, 27.5, and 13.75 µM), and the results show that the combination of an AKT inhibitor and lysine reduction significantly enhanced the cytostatic effect in *EGFR*-mutant NSCLC cell lines (Figure 7A for PC9, Figure 7B for NCI-H1975, Figure 7C for HCC827, and Figure 7D for HCC4006). These results indicate that the upregulation of phosphor-AKT plays a pro-survival role in the context of lysine reduction in *EGFR*-mutant NSCLC cells. We consistently used osimertinib to suppress *EGFR*-mutant cell lines with *EGFR*-mutant cell lines under lysine reduction, and the results showed that this treatment in along with lysine reduction (55, 27.5, and 13.75 µM) can enhance osimertinib-induced cytotoxicity in a concentration-dependent manner in *EGFR*-mutant cells (Figure 7E for PC9, Figure 7F for NCI-H1975, Figure 7G for HCC827, and Figure 7H for HCC4006).

## 3. Discussion

In this study, we demonstrate, for the first time, the effectiveness of applying lysine reduction to *EGFR*-mutant NSCLC cells as a potential therapeutic strategy. We found that *EGFR*-mutant NSCLC cells, including PC9, NCI-H1975, HCC827, HCC4006, and NCI-H3255, were highly sensitive to lysine deprivation. In addition, lysine deprivation was able to reduce the survival of PC9 sublines that were resistant to second- (afatinib) and third-generation (osimertinib) EGFR-TKIs. We examined the genes involved in lysine catabolism, including the saccharopine and pipecolate pathways, and found that the expression of *AADAT* consistently increased in response to lysine deprivation in all *EGFR*-mutant NSCLC cells, and that this occurred alongside the induction of phosphor-AKT for survival in *EGFR*-mutant NSCLC cells. Moreover, we determined that *AADAT* expression can be regulated by EGFR–AKT signaling in *EGFR*-mutant NSCLC cells. Finally, we found that a combination of osimertinib and lysine reduction can enhance the cytostatic effect of single-agent osimertinib in *EGFR*-mutant NSCLC cells.

Various studies have demonstrated that gene alterations can make cancer cells highly sensitive to stresses related to specific amino acids, including EAAs and NEAAs [26]. Increasing evidence has shown that the induction of stress based on a particular amino acid might be a strategy to target cancer cells; however, cancer cells could have differing abilities to synthesize NEAAs, which might confound the therapeutic targets of particular cancer types [20]. Indeed, in the present study, we found that all *EGFR*-mutant NSCLC cells were inconsistently sensitive to the short-term deprivation of NEAA (Asp, Asn, Glu, and Ser) (Figure 1 and Figure 2). In addition, our results show that some *EGFR*-mutant NSCLC cells were sensitive to arginine deprivation (conditional EAAs), consistent with the previous findings in NSCLC cells [27]. Nevertheless, some *EGFR*-mutant NSCLC cells were insensitive or partially sensitive to arginine deprivation (Figure 1). In addition, glutamine (conditional EAAs) is known to be essential to cancer cells [28], and the glutamine-deprivation-induced cytostatic effect was inconsistent among the different *EGFR*-mutant NSCLC cells (Figure 1). In contrast to NEAAs and conditional EAAs, we found that all *EGFR*-mutant NSCLC cells were consistently sensitive to lysine deprivation, which is an EAA (Figure 1 and Figure 3). These results confirmed the previous findings for other types of cancers that particular gene alterations can sensitize cancer cells to specific EAA stress [20,21,22,23,24]. In addition, our results indicate that short-term lysine deprivation would not significantly affect the survival of *EGFR* wild-type NSCLC and normal cells (Figure 1E), which is consistent with reports from previous studies on normal cells and other types of cancers [20,29,30]. These findings suggest that gene alteration in *EGFR* specifically makes *EGFR*-mutant NSCLC cells to be more dependent on lysine for cellular viability.

Drug-induced amino acid deprivation provides an attainable improvement in anticancer treatment with increased therapeutic efficacy and fewer adverse side effects [31]. For instance, a previous study revealed that the depletion of methionine could sensitize triple-negative breast cancer cells to lexatumumab treatment [21]. In addition, the combination of arginine depletion and chloroquine significantly caused sarcoma cell death in vitro and in vivo [32]. As shown in Figure 7, we predominantly observed a robust reduction in the survival of *EGFR*-mutant NSCLC cells under AKT inhibitor/osimertinib treatment in combination with lysine reduction. Our analysis demonstrates that the induction of lysine stress might be an excellent strategy to enhance the therapeutic effects of EGFR-TKIs in *EGFR*-mutant NSCLC. EGFR-TKIs are effective against NSCLC by activating *EGFR* mutations; however, acquired resistance always inevitably occurs. Hence, there is an urgent need to find a way to treat this resistance. In the present study, we found that the afatinib-resistant subline (PC9-AR-C3) (Figure 2A, Figures S2 and S3) and osimertinib-resistant sublines (PC9-OR-D4 and PC9-OR-E8) (Figure 2B, Figures S4 and S5) were sensitive to the short-term depletion of lysine (Figure 3). Several recent studies have suggested that a diet involving the restriction of specific amino acids could be used against particular types of cancer to precisely reduce the rate of tumor growth and metastasis [33,34]. In addition, recent research using patient-derived xenograft (PDX) models demonstrated that the manipulation of dietary methionine restriction might be a strategy to improve the outcomes of therapy for colorectal cancer [35]. We propose that the development of drugs able to induce lysine stress and offer limited lysine uptake could benefit *EGFR*-mutant NSCLC patients with resistance to EGFR-TKIs.

In human lysine catabolism, the AADAT enzyme acts in the concluding step of the saccharopine pathway, transforming L-lysine to α-ketoadipic acid [36,37]; how *AADAT* is regulated is still unclear. In this study, we found a consistent effect of lysine deprivation in the induction of *AADAT* expression in all *EGFR*-mutant and EGFR-TKI resistant NSCLC cells (Figure 4, Figures S3B and S5B,D). The up-regulation of *AADAT* expression might be the complementary response for the deficiency of lysine in *EGFR*-mutant and EGFR-TKI resistant NSCLC cells. Our results also show that this induction occurred concomitant with the upregulation of phospho-AKT in all *EGFR*-mutant and EGFR-TKI resistant NSCLC cells (Figure 5, Figures S3C and S5C,E). In addition, our study revealed that the expression of *AADAT* could be regulated by EGFR–AKT signaling in *EGFR*-mutant NSCLC cells (Figure 6). Interestingly, we also found that the upregulation of phospho-AKT appeared to be an adaptive pro-survival of *EGFR*-mutant NSCLC cells under lysine reduction (Figure 7), which is consistent with the pro-survival role of AKT observed under other stresses in cancer cells [38,39]. AKT is a protein kinase that regulates numerous transcriptional factors, including TP53, FOXOs, and CREB, which are important in cancer metabolism [40,41,42]. Therefore, understanding the involvement of transcriptional factors in the mechanism by which AKT regulates the expression of the AADAT gene in *EGFR-*mutant NSCLC cells is worthy of further investigation.

EGFR signaling is continuously activated in *EGFR*-mutant NSCLC and moderates the phosphorylation of several downstream protein kinases, including AKT. In our study, we investigated how EGFR–AKT signaling regulates AADAT (Figure 6). Under normal conditions, we used EGFR-TKIs to block the phosphorylation of EGFR downstream signaling and an AKT inhibitor to restrain AKT phosphorylation (Figure 6A). We ultimately found a significant reduction in *AADAT* gene expression. Furthermore, we established an afatinib-resistant clone (PC9-AR-C3) (Figure 2A) with an EGFR-T790M mutation (Figure S2). It is well known that the T790M mutation impacts the binding of first-generation (gefitinib) and second-generation (afatinib) EGFR-TKIs, which influences their effectiveness. Thus, under the treatment of gefitinib and afatinib, PC9-AR-C3 showed no reduction in *AADAT* gene expression. Osimertinib (third-generation EGFR-TKI), however, not only targets EGFR harboring activating mutations (L858R and exon-19-deletion) but also the T790M mutation [10,11,12]. We demonstrated that osimertinib can effectively inhibit AADAT gene expression when compared with the gefitinib and afatinib treatment groups (Figure 6D). Moreover, we further examined both osimertinib-resistant sublines (PC9-OR-D4 and PC9-OR-E8) (Figure 2B) and discovered that osimertinib is unable to inhibit the phosphorylation of AKT (Figure S5) and the induction of *AADAT* gene expression (Figure 6E). These results demonstrate that the phosphorylation of AKT is moderated by EGFR signaling in *EGFR*-mutant NSCLC. Nevertheless, Sanger sequencing of PC9-OR-D4 and PC9-OR-E8 revealed they did not carry the C797S mutation (Figure S4), which is an acquired mutation that impairs the binding efficiency of osimertinib to EGFR mutants [43]. Several EGFR–independent mechanisms, including *BRAF*-V600E, *KRAS* mutations, oncogenic fusions (NTRK-, RET-, ALK-fusion), *MET*- and *HER2*-amplification/mutation, epithelial-to-mesenchymal transition (EMT), and cell type transformation (from NSCLC to SCLC) were reported to confer resistance to osimertinib [25,44,45]. In our study, we found NCI-H1975 cells harboring exon-16-skipping HER2, which is known to be an osimertinib-resistant cell line [22], were also sensitive to short-term lysine deprivation (Figure S5). Therefore, it is worthy to further examine whether the different type of lung cancer (e.g., SCLC) or NSCLC harboring other genetic mutations for osimertinib-resistance can also lead sensitize NSCLC cells to lysine deprivation. Moreover, we found that AKT phosphorylation and *AADAT* gene expression are increased under lysine deprivation without any changes in EGFR phosphorylation (Figure 5). This result indicates that under lysine depletion conditions, the phosphorylation of AKT in *EGFR*-mutant NSCLC might be controlled not only by EGFR signaling but also by adaptive responses. The upregulation of AKT phosphorylation may have resulted from cross-talk signaling (such as between AMPK and PTEN)**,** upstream kinase activation (such as PDK1, mTOR complex 2, and CDK2), and phosphatase inactivation (including PP2A and PHLPP) [46,47,48]. It would be worthwhile to investigate the adaptive responses to lysine deprivation besides EGFR signaling in *EGFR*-mutant NSCLC.

## 4. Materials and Methods

### 4.1. Reagents and Antibodies

Osimertinib (AZD9291) was provided by AstraZeneca Pharmaceuticals (Cambridge, UK). Afatinib (BIBW 2992) was provided by Boehringer Ingelheim (Ingelheim, Germany). Gefitinib and MK2206 were purchased from Selleck (Houston, TX, USA). All drugs were dissolved in dimethyl sulfoxide (DMSO) to create stocks at a concentration of 10 mM and stored in aliquots at −80 °C. The L-amino acid kit was purchased from Sigma-Aldrich (St. Louis, MO, USA). Antibodies against phosphor-EGFR (Y1068), EGFR, phospho-AKT (S473), AKT, phospho-ERK (T202/Y204), and ERK were purchased from Cell Signaling Technologies (Beverly, MA, USA). Antibodies against α-tubulin were purchased from Sigma-Aldrich.

### 4.2. Cell Culture

The human *EGFR*-mutant (exon-19 deletion: HCC4006, HCC827, and PC9; L858R: NCI-H3255; L858R/T790M: NCI-H1975), *EGFR* wild-type (NCI-H441, NCI-H838, NCI-H838-*KRAS*^G12V^, and NCI-H838-*KRAS*^G12D^) NSCLC cell lines, normal lung fibroblast cell line (MRC-5), and rat astrocyte cell line (CTX TNA2) were maintained in RPMI-1640 medium (Gibco, Waltham, MA, USA) supplemented with 10% fetal bovine serum (FBS), 2 mM L-glutamine, and an antibiotic–antimycotic solution consisting of 100 units/mL penicillin G, 100 μg/mL streptomycin sulfate, and 250 ng/mL amphotericin B. The human NCI-H1975 stable expression of exon-16-skipping HER2 (HERD16) was cultured under the same conditions as above and supplemented with G418 solution (600 μg/mL). Cells were incubated in a humidified atmosphere of 5% CO_2_ at 37 °C.

### 4.3. Establishment of EGFR-TKI-Resistant Clones

To establish the EGFR-TKI-resistant clones, EGFR-TKIs were added at an initial dose of half-maximal inhibitory concentration (IC50) (0.5 nM for afatinib and 5 nM for osimertinib), and the cells were cultured in drug-containing RPMI-1640 medium. The dosage of EGFR-TKIs was doubled after cells had reached a high confluency, and this was carried out twice. When the dosage reached 1 μM, the cells were split via single-cell analysis, and resistant clones were selected. The IC50 for the EGFR-TKI of each resistant clone was validated using sulforhodamine B (SRB) assay.

### 4.4. Sulforhodamine B (SRB) Cell Viability Assay

SRB assay was used to determine cell viability [49], which depended on the measured cellular protein content. The cells were seeded in 96-well plates in triplicate for each condition. After treatment with drugs for 96 h, the cells were fixed using 10% ice-cold trichloroacetic acid (TCA) (Sigma-Aldrich) at 4 °C for 1 h, rinsed four times with distilled water, and air-dried. The cells were then stained with 0.057% SRB (Sigma-Aldrich) in 1% acetic acid for 30 min at 24 °C. After washing four times with 1% acetic acid and being air-dried, 200 µL of 10 mM Tris-base (pH 10.5) was added into each well and incubated for 30 min at 24 °C. The colorimetric readings were performed using a microplate reader at 540 nm.

### 4.5. Screening Platform of Amino Acid Stresses

RPMI-1640-based media with the entire amino acid solution and the media deprived of each amino acid were prepared using 7.4 g/L of basal RPMI-1640 medium powder (US Biological Life Sciences, Swampscott, MA, USA) by adding 2 g/L of D-glucose, 2 g/L of sodium bicarbonate, and either all 20 amino acids (for the control RPMI-1640 medium) or 19 amino acids (for the deprivation of one amino acid). The concentration of each amino acid used is described in Table A2. All media were supplemented with 10% FBS, 2 mM L-glutamine (except for the glutamine-deprived medium), and an antibiotic–antimycotic solution consisting of 100 units/mL penicillin G, 100 μg/mL streptomycin sulfate, and 250 ng/mL amphotericin B. The sensitivities of the cancer cells to each type of amino acid deprivation were determined using an SRB assay.

### 4.6. RNA Extraction

Total RNA was extracted from the samples using Direct-zol RNA MicroPrep (Zymo Research, Irvine, CA, USA) according to the manufacturer’s instructions. TRIzol reagent (Zymo Research, Irvine, CA, USA) was used to lyse the cells at each time point of the experiment. After centrifugation at 12,000× *g* for 1 min at 4 °C, the clear supernatant was collected and incubated with DNase I for 15 min at 24 °C. Next, we transferred the mixture into a Zymo-Spin™ IC column in collection tubes and centrifuged the mixture at 12,000× *g* for 1 min at 4 °C. The RNA wash buffer was washed twice, and the RNA was eluted with 40 μL of RNase-free water.

### 4.7. Real-Time PCR Analysis

The cDNA was obtained using a PrimeScript RT Reagent Kit (Clontech, Kusatsu, Shiga, Japan) according to the manufacturer’s instructions. Gene expression levels were quantified using real-time qPCR. The PCR reaction was performed in a StepOne™ Real-Time PCR system (Life Technologies, Carlsbad, CA, USA) using KAPA SYBR FAST qPCR Kits (Kapa Biosystems, Wilmington, MA, USA). All primer sets used in this study are described in the Table A1. The reaction mixture was first denatured at 95 °C for 3 min. The PCR conditions were 95 °C for 3 s and 60 °C for 30 s for 40 cycles. Gene expression levels were calculated using the 2^−ΔΔCt^ method.

### 4.8. Western Blot Analysis

At the appropriate time point for each experiment, whole-cell lysates were prepared using a radioimmunoprecipitation assay (RIPA) buffer (Cell Signaling Technologies, Beverly, MA, USA) with a cocktail of phosphatase inhibitors (Thermo Fisher Scientific, San Jose, CA, USA). The protein concentrations were determined using a Bradford assay (Sigma-Aldrich, St. Louis, MO, USA). For the Western blot analysis (reducing system), the samples were diluted in 5× Laemmli buffer (300 mM Tris-HCl pH 6.8, 10% SDS (*w*/*v*), 5%, 2-mercaptoethanol, 25% glycerol (*v*/*v*), and 0.1% bromophenol blue (*w/v*)) and boiled for 5 min. For the non-reducing PAGE conditions, the samples were diluted in 5X Laemmli buffer without 2-mercaptoethanol and boiled for 5 min. Proteins (20 μg) were separated using 8–15% SDS-PAGE and transferred onto polyvinylidene fluoride (PVDF) membranes (GE Healthcare Life Sciences, Madison, WI, USA). The nonspecific binding sites on the PVDF membranes were blocked with 5% non-fat milk in TBST (20 mM Tris-HCl, pH 7.6, 137 mM NaCl, 1% Tween-20). The membranes were then hybridized with primary antibodies overnight at 4 °C, followed by incubation with horseradish peroxidase (HRP)-conjugated secondary antibodies for 1 h at 24 °C. The membranes were then developed using Immobilon Western Chemiluminescence HRP substrates (Merck Millipore, Billerica, MA, USA).

### 4.9. Statistical Analysis

All data are presented as the mean ± SEM. Statistical differences between the control and treated groups were determined using Student’s *t*-test, and the results were considered statistically significant at *p* < 0.05.

## 5. Conclusions

Our investigations indicated that NSCLC with the *EGFR* mutation is sensitive to the availability of lysine. Lysine deprivation diminished the survival and affected the morphology of *EGFR*-mutant NSCLC cells. Accordingly, AADAT, an enzyme involved in lysine catabolism, can be regulated by EGFR–AKT signaling in *EGFR*-mutant NSCLC cells. Lastly, lysine deficiency was able to overcome the resistance of EGFR-TKIs and vigorously reduced cell survival under a combination of osimertinib-treatment in *EGFR*-mutant NSCLC cells.

## Data Availability

Data is contained within the article or supplementary material.

## Supplementary Materials

The following are available online at https://www.mdpi.com/2072-6694/13/2/272/s1, Figure S1: The results of PCR and DNA sequencing on the identification of G12D and G12V point mutations in NCI-H838^G12D^ and NCI-H838^G12V^. Figure S2: The results of DNA sequencing for the identification of EGFRT790M-positive mutations in exon 20 in the PC9 afatinib-resistant subline (PC9-AR-C3). Figure S3: Acquired resistance under afatinib treatment and the impact of lysine deprivation in AADAT and p-AKT/AKT signaling in the PC9 afatinib-resistant clone. Figure S4: The results of DNA sequencing of exon 20 in the PC9 osimertinib-resistant sublines. Figure S5: Acquired resistance under osimertinib treatment and the impact of lysine deprivation on AADAT and p-AKT/AKT signaling in the PC9 osimertinib-resistant clone. Figure S6: The survival of NCI-H1975-HER2D16 cell lines under lysine deprivation. Figure S7: The original Western blot films for Figure 5 and Figure 6. Figure S8: The original Western blot films for Figures S3 and S5.

## Appendix A

**Table A1:** Components of control and basal RPMI media allowing for comparison of differences.

Components (Code)	Normal RPMI-1640 (mM)	Basal RPMI-1640
Glycine (G)	0.13333334	Absent
L-Arginine (R)	1.1494253	Absent
L-Asparagine (N)	0.37878788	Absent
L-Aspartic acid (D)	0.15037593	Absent
L-Cystine hydrochloride (C)	0.20766774	Absent
L-Glutamic Acid (E)	0.13605443	Absent
L-Glutamine (Q)	2.0547945	Absent
L-Histidine (H)	0.09677419	Absent
L-Hydroxyproline (O)	0.15267175	Absent
L-Isoleucine (I)	0.3816794	Absent
L-Leucine (L)	0.3816794	Absent
L-Lysine hydrochloride (K)	0.21857923	Absent
L-Methionine (M)	0.10067114	Absent
L-Phenylalanine (F)	0.09090909	Absent
L-Proline (P)	0.17391305	Absent
L-Serine (S)	0.2857143	Absent
L-Threonine (T)	0.16806723	Absent
L-Tryptophan (W)	0.024509804	Absent
L-Tyrosine disodium salt dihydrate (Y)	0.11111111	Absent
L-Valine (V)	0.17094018	Absent
D-Glucose	11.111111	Absent

**Table A2:** The primer sets used in this study.

Primer Name	Sequence (5′ to 3′)	Note
*AASS*-Forward	ATG GGA CGG TGT TAA GTC GT	Lysine catabolism
*AASS*-Reverse	CAT CTT GGC GGG TTA GGA GG	Lysine catabolism
*ALDH7A1*-Forward	GAG GAA AAC GAG GGC GTG TA	Lysine catabolism
*ALDH7A1*-Reverse	GAT CTT CTC CCG CAA GGC AT	Lysine catabolism
*AADAT*-Forward	TTG GCT GGT GGC TTA CCA AA	Lysine catabolism
*AADAT*-Reverse	TGT CCT TGA CTG GGT GGG TA	Lysine catabolism
*DHKTD1*-Forward	AGG CAT GCA ATC GTG GTT TG	Lysine catabolism
*DHKTD1*-Reverse	TGG GCT CTC AAT GCT CAT CC	Lysine catabolism
*KYAT3*-Forward	TAG CGG TAG AGC AAA ATT CCT GA	Lysine catabolism
*KYAT3*-Reverse	GGC CAA GAT TCA CAA CAG AAG G	Lysine catabolism
*CRYM*-Forward	GGT CTG TTC TTC GGT CCA GG	Lysine catabolism
*CRYM*-Reverse	GGG AAT CCA CGT ACA GCA CA	Lysine catabolism
*PIPOX*-Forward	TCA ACG TGT GTT ACT GGC GA	Lysine catabolism
*PIPOX*-Reverse	CAG TCC GTA GAT GTG GTG GG	Lysine catabolism
*GAPDH*-Forward	GGA GCG AGA TCC CTC CAA AAT	Lysine catabolism
*GAPDH*-Reverse	GGC TGT TGT CAT ACT TCT CAT GG	Lysine catabolism
*KRAS*-IF2	ACG TCT GCA GTC AAC TGG AAT	*KRAS* exon 2
*KRAS*-IR2	AGA ATG GTC CTG CAC CAG TAA	*KRAS* exon 2
*EGFR*-IF20	GAA ACT CAA GAT CGC ATT CAT GC	T790M validation
*EGFR*-IR20	GCA AAC TCT TGC TAT CCC AGG AG	T790M validation

## References

[1] Rosell R., Moran T., Queralt C., Porta R., Cardenal F., Camps C., Majem M., Lopez-Vivanco G., Isla D., Provencio M. (2009). Screening for epidermal growth factor receptor mutations in lung cancer. N. Engl. J. Med..

[2] Shi Y., Au J.S., Thongprasert S., Srinivasan S., Tsai C.M., Khoa M.T., Heeroma K., Itoh Y., Cornelio G., Yang P.C. (2014). A prospective, molecular epidemiology study of EGFR mutations in Asian patients with advanced non-small-cell lung cancer of adenocarcinoma histology (PIONEER). J. Thorac. Oncol..

[3] da Cunha Santos G., Shepherd F.A., Tsao M.S. (2011). EGFR mutations and lung cancer. Annu. Rev. Pathol..

[4] Mok T.S., Wu Y.L., Thongprasert S., Yang C.H., Chu D.T., Saijo N., Sunpaweravong P., Han B., Margono B., Ichinose Y. (2009). Gefitinib or carboplatin-paclitaxel in pulmonary adenocarcinoma. N. Engl. J. Med..

[5] Lee C.K., Brown C., Gralla R.J., Hirsh V., Thongprasert S., Tsai C.M., Tan E.H., Ho J.C., Chu da T., Zaatar A. (2013). Impact of EGFR inhibitor in non-small cell lung cancer on progression-free and overall survival: A meta-analysis. J. Natl. Cancer Inst..

[6] Miller V.A., Hirsh V., Cadranel J., Chen Y.M., Park K., Kim S.W., Zhou C., Su W.C., Wang M., Sun Y. (2012). Afatinib versus placebo for patients with advanced, metastatic non-small-cell lung cancer after failure of erlotinib, gefitinib, or both, and one or two lines of chemotherapy (LUX-Lung 1): A phase 2b/3 randomised trial. Lancet Oncol..

[7] Wu Y.L., Zhou C., Hu C.P., Feng J., Lu S., Huang Y., Li W., Hou M., Shi J.H., Lee K.Y. (2014). Afatinib versus cisplatin plus gemcitabine for first-line treatment of Asian patients with advanced non-small-cell lung cancer harbouring EGFR mutations (LUX-Lung 6): An open-label, randomised phase 3 trial. Lancet Oncol..

[8] Park K., Wan-Teck Lim D., Okamoto I., Yang J.C. (2019). First-line afatinib for the treatment of EGFR mutation-positive non-small-cell lung cancer in the ‘real-world’ clinical setting. Ther. Adv. Med. Oncol..

[9] Rotow J., Bivona T.G. (2017). Understanding and targeting resistance mechanisms in NSCLC. Nat. Rev. Cancer.

[10] Cross D.A., Ashton S.E., Ghiorghiu S., Eberlein C., Nebhan C.A., Spitzler P.J., Orme J.P., Finlay M.R., Ward R.A., Mellor M.J. (2014). AZD9291, an irreversible EGFR TKI, overcomes T790M-mediated resistance to EGFR inhibitors in lung cancer. Cancer Discov..

[11] Janne P.A., Yang J.C., Kim D.W., Planchard D., Ohe Y., Ramalingam S.S., Ahn M.J., Kim S.W., Su W.C., Horn L. (2015). AZD9291 in EGFR inhibitor-resistant non-small-cell lung cancer. N. Engl. J. Med..

[12] Yang J.C., Ahn M.J., Kim D.W., Ramalingam S.S., Sequist L.V., Su W.C., Kim S.W., Kim J.H., Planchard D., Felip E. (2017). Osimertinib in pretreated T790M-positive advanced non-small-cell lung cancer: AURA study phase II extension component. J. Clin. Oncol..

[13] Lyssiotis C.A., Kimmelman A.C. (2017). Metabolic interactions in the tumor microenvironment. Trends Cell Biol..

[14] Anastasiou D. (2017). Tumour microenvironment factors shaping the cancer metabolism landscape. Br. J. Cancer.

[15] Aliu E., Kanungo S., Arnold G.L. (2018). Amino acid disorders. Ann. Transl. Med..

[16] Knerr I. (2016). Chapter 21-Amino Acid-Related Diseases. Mol. Nutr. Amino Acids Proteins.

[17] Finicle B.T., Jayashankar V., Edinger A.L. (2018). Nutrient scavenging in cancer. Nat. Rev. Cancer.

[18] Vettore L., Westbrook R.L., Tennant D.A. (2020). New aspects of amino acid metabolism in cancer. Br. J. Cancer.

[19] Lieu E.L., Nguyen T., Rhyne S., Kim J. (2020). Amino acids in cancer. Exp. Mol. Med..

[20] Sheen J.H., Zoncu R., Kim D., Sabatini D.M. (2011). Defective regulation of autophagy upon leucine deprivation reveals a targetable liability of human melanoma cells in vitro and in vivo. Cancer Cell.

[21] Strekalova E., Malin D., Good D.M., Cryns V.L. (2015). Methionine deprivation induces a targetable vulnerability in triple-negative breast cancer cells by enhancing TRAIL Receptor-2 expression. Clin. Cancer Res..

[22] Mayers J.R., Torrence M.E., Danai L.V., Papagiannakopoulos T., Davidson S.M., Bauer M.R., Lau A.N., Ji B.W., Dixit P.D., Hosios A.M. (2016). Tissue of origin dictates branched-chain amino acid metabolism in mutant Kras-driven cancers. Science.

[23] Ohtawa K., Ueno T., Mitsui K., Kodera Y., Hiroto M., Matsushima A., Nishimura H., Inada Y. (1998). Apoptosis of leukemia cells induced by valine-deficient medium. Leukemia.

[24] Woolley P.V., Dion R.L., Bono V.H. (1974). Effects of tryptophan deprivation on L1210 cells in culture. Cancer Res..

[25] Hsu C.C., Liao B.C., Liao W.Y., Markovets A., Stetson D., Thress K., Yang J.C. (2020). Exon 16-Skipping HER2 as a novel mechanism of osimertinib resistance in EGFR L858R/T790M-positive non-small cell lung cancer. J. Thorac. Oncol..

[26] Lukey M.J., Katt W.P., Cerione R.A. (2017). Targeting amino acid metabolism for cancer therapy. Drug Disc. Today.

[27] Shen W., Zhang X., Fu X., Fan J., Luan J., Cao Z., Yang P., Xu Z., Ju D. (2017). A novel and promising therapeutic approach for NSCLC: Recombinant human arginase alone or combined with autophagy inhibitor. Cell Death Dis..

[28] Altman B.J., Stine Z.E., Dang C.V. (2016). From Krebs to clinic: Glutamine metabolism to cancer therapy. Nat. Rev. Cancer.

[29] Tang X., Wu J., Ding C.K., Lu M., Keenan M.M., Lin C.C., Lin C.A., Wang C.C., George D., Hsu D.S. (2016). Cystine deprivation triggers programmed necrosis in VHL-deficient renal cell carcinomas. Cancer Res..

[30] Tang X., Ding C.K., Wu J., Sjol J., Wardell S., Spasojevic I., George D., McDonnell D.P., Hsu D.S., Chang J.T. (2017). Cystine addiction of triple-negative breast cancer associated with EMT augmented death signaling. Oncogene.

[31] Fung M.K.L., Chan G.C. (2017). Drug-induced amino acid deprivation as strategy for cancer therapy. J. Hematol. Oncol..

[32] Bean G.R., Kremer J.C., Prudner B.C., Schenone A.D., Yao J.C., Schultze M.B., Chen D.Y., Tanas M.R., Adkins D.R., Bomalaski J. (2016). A metabolic synthetic lethal strategy with arginine deprivation and chloroquine leads to cell death in ASS1-deficient sarcomas. Cell Death Dis..

[33] Muthusamy T., Cordes T., Handzlik M.K., You L., Lim E.W., Gengatharan J., Pinto A.F.M., Badur M.G., Kolar M.J., Wallace M. (2020). Serine restriction alters sphingolipid diversity to constrain tumour growth. Nature.

[34] Knott S.R.V., Wagenblast E., Khan S., Kim S.Y., Soto M., Wagner M., Turgeon M.O., Fish L., Erard N., Gable A.L. (2018). Asparagine bioavailability governs metastasis in a model of breast cancer. Nature.

[35] Gao X., Sanderson S.M., Dai Z., Reid M.A., Cooper D.E., Lu M., Richie J.P., Ciccarella A., Calcagnotto A., Mikhael P.G. (2019). Dietary methionine influences therapy in mouse cancer models and alters human metabolism. Nature.

[36] Hallen A., Jamie J.F., Cooper A.J. (2013). Lysine metabolism in mammalian brain: An update on the importance of recent discoveries. Amino Acids.

[37] Goh D.L., Patel A., Thomas G.H., Salomons G.S., Schor D.S., Jakobs C., Geraghty M.T. (2002). Characterization of the human gene encoding alpha-aminoadipate aminotransferase (AADAT). Mol. Genet. Metab..

[38] Gao M., Liang J., Lu Y., Guo H., German P., Bai S., Jonasch E., Yang X., Mills G.B., Ding Z. (2014). Site-specific activation of AKT protects cells from death induced by glucose deprivation. Oncogene.

[39] Rasool R.U., Nayak D., Chakraborty S., Faheem M.M., Rah B., Mahajan P., Gopinath V., Katoch A., Iqra Z., Yousuf S.K. (2017). AKT is indispensable for coordinating Par-4/JNK cross talk in p21 downmodulation during ER stress. Oncogenesis.

[40] Eijkelenboom A., Burgering B.M. (2013). FOXOs: Signalling integrators for homeostasis maintenance. Nat. Rev. Mol. Cell. Biol..

[41] Vousden K.H., Ryan K.M. (2009). p53 and metabolism. Nat. Rev. Cancer.

[42] Altarejos J.Y., Montminy M. (2011). CREB and the CRTC co-activators: Sensors for hormonal and metabolic signals. Nat. Rev. Mol. Cell Biol..

[43] Thress K.S., Paweletz C.P., Felip E., Cho B.C., Stetson D., Dougherty B., Lai Z., Markovets A., Vivancos A., Kuang Y. (2015). Acquired EGFR C797S mutation mediates resistance to AZD9291 in non-small cell lung cancer harboring EGFR T790M. Nat. Med..

[44] Leonetti A., Sharma S., Minari R., Perego P., Giovannetti E., Tiseo M. (2019). Resistance mechanisms to osimertinib in EGFR-mutated non-small cell lung cancer. Br. J. Cancer.

[45] Lin C.C., Shih J.Y., Yu C.J., Ho C.C., Liao W.Y., Lee J.H., Tsai T.H., Su K.Y., Hsieh M.S., Chang Y.L. (2018). Outcomes in patients with non-small-cell lung cancer and acquired Thr790Met mutation treated with osimertinib: A genomic study. Lancet Respir. Med..

[46] Zhao Y., Hu X., Liu Y., Dong S., Wen Z., He W., Zhang S., Huang Q., Shi M. (2017). ROS signaling under metabolic stress: Cross-talk between AMPK and AKT pathway. Mol. Cancer.

[47] Manning B.D., Toker A. (2017). AKT/PKB Signaling: Navigating the Network. Cell.

[48] Jung K.J., Kim D.H., Lee E.K., Song C.W., Yu B.P., Chung H.Y. (2013). Oxidative stress induces inactivation of protein phosphatase 2A, promoting proinflammatory NF-κB in aged rat kidney. Free Radic. Biol. Med..

[49] Vichai V., Kirtikara K. (2006). Sulforhodamine B colorimetric assay for cytotoxicity screening. Nat. Protoc..

