# Supplementary material for: Lysine Deprivation Induces AKT-AADAT Signaling and Overcomes EGFR-TKIs Resistance in EGFR-Mutant Non-Small Cell Lung Cancer Cells
Source: Cancers (Basel). 2021 Jan 13;13(2):272. doi: 10.3390/cancers13020272 (PMC7828377; doi:10.3390/cancers13020272)
Supplement: Supplementary file 1 [file cancers-13-00272-s001.pdf]

cancers

MDPI

Supplementary Materials: Lysine Deprivation Induces AKT-AADAT Signaling and Overcomes EGFR-TKIs Resistance in EGFR-Mutant Non-Small Cell Lung Cancer Cells

Chia-Chi Hsu, Albert Ying-Po Yang, Jui-Yi Chen, Hsin-Hui Tsai, Shu-Heng Lin, Pei-Chen Tai, Ming-Hung Huang, Wei-Hsun Hsu, Anya Maan-Yuh Lin and James Chih-Hsin Yang

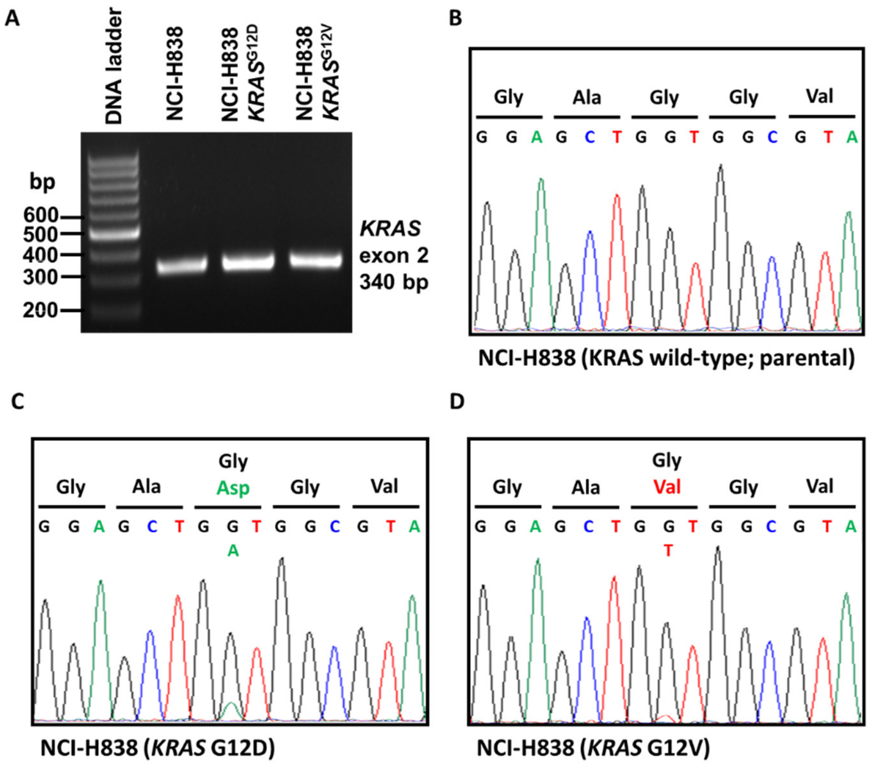

Figure S1. The results of PCR and DNA sequencing for the identification of G12D and G12V point mutations in NCI-H838^{G12D} and NCI-H838^{G12V}. (**A**) Genomic DNA was extracted from NCI-H838, NCI-H838-*KRAS* (G12D), and NCI-H838-*KRAS* (G12V) cells, and the partial region of exon 2 of the *KRAS* gene was amplified using PCR with the primer set described in Appendix A (Table A1). The point mutation on the amino acid of *KRAS* in position 12 was verified using Sanger sequencing. (**B**) wild type; (**C**) G12D; (**D**) G12V.

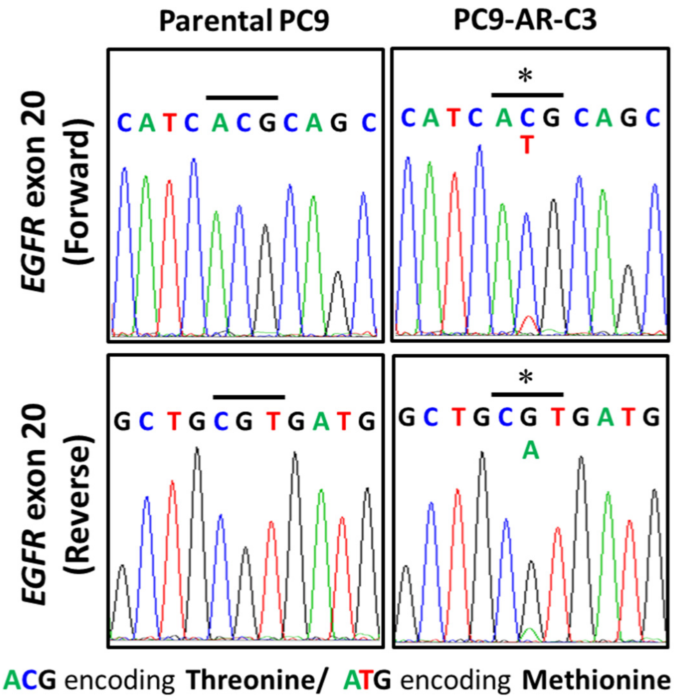

Figure S2. The results of DNA sequencing for the identification of *EGFR*^{T790M}-positive mutations in exon 20 in the PC9 afatinib-resistant subline (PC9-AR-C3). Genomic DNA was extracted from both parental PC9 cell lines and PC9-AR-C3, and the T790M mutation on *EGFR* was confirmed by Sanger sequencing of exon 20 in the *EGFR* gene (* $p < 0.05$).

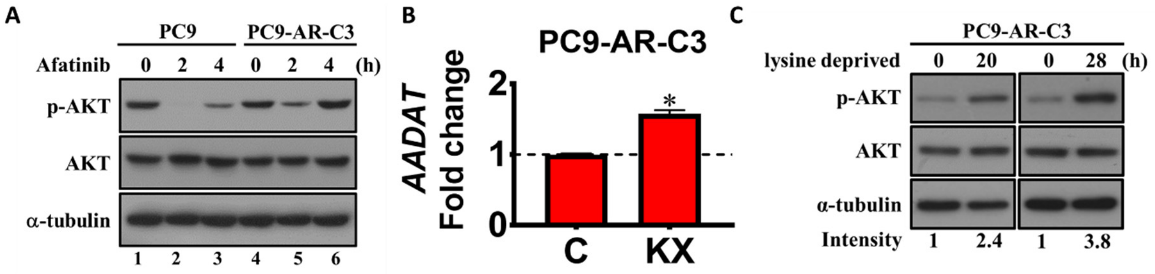

Figure S3. Acquired resistance under afatinib treatment and the impact of lysine deprivation in AADAT and p-AKT/AKT signaling in the PC9 afatinib-resistant clone. (**A**) The PC9 and afatinib-resistant clone (PC9-AR-C3) were treated with afatinib (3 nM) for 0, 2, and 4 h, and whole-cell extracts were used for Western blot analysis. The Western blot analysis was performed using antibodies against phosphor-AKT (p-AKT), AKT, and α-tubulin. (**B**) PC9-AR-C3 was incubated with either the lysine-deprived (0 μM lysine) or control medium (220 μM lysine) for 24 h. The expression of genes involved in the lysine catabolism was analyzed using qPCR. The relative fold change in the expression level of each gene in each lysine-deprived group was normalized to the corresponding control. The results shown are representative of three independent experiments. C, normal RPMI; KX, lysine-deprived RPMI; AADAT, kynurenine/α-aminoadipate aminotransferase. The data are presented as the mean ± SEM from three independent experiments (*, $p < 0.05$, compared with the normal RPMI group). (**C**) PC9-AR-C3 was incubated with lysine-deprived RPMI for 20 and 28 h, and whole-cell extracts were used for the Western blot analysis with antibodies against phosphor-AKT (p-AKT), AKT, and α-tubulin. The Western blot analysis of each relative fold change was measured using the ImageJ software, normalized to each control group. Western blot analyses were performed using antibodies against phosphor-AKT (p-AKT), AKT, and α-tubulin. Uncropped Western Blots can be found in Figure S8.

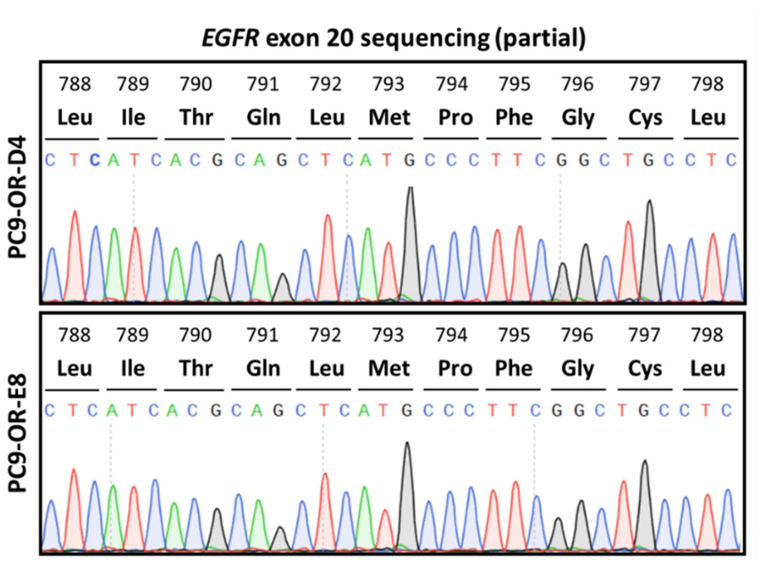

Figure S4. The results of DNA sequencing of exon 20 in the PC9 osimertinib-resistant sublines. Genomic DNA was extracted from both parental PC9-OR-D4 and PC9-OR-E8, and the mutational status of T790 and C797 on *EGFR* were confirmed by Sanger sequencing of exon 20 in the *EGFR* gene.

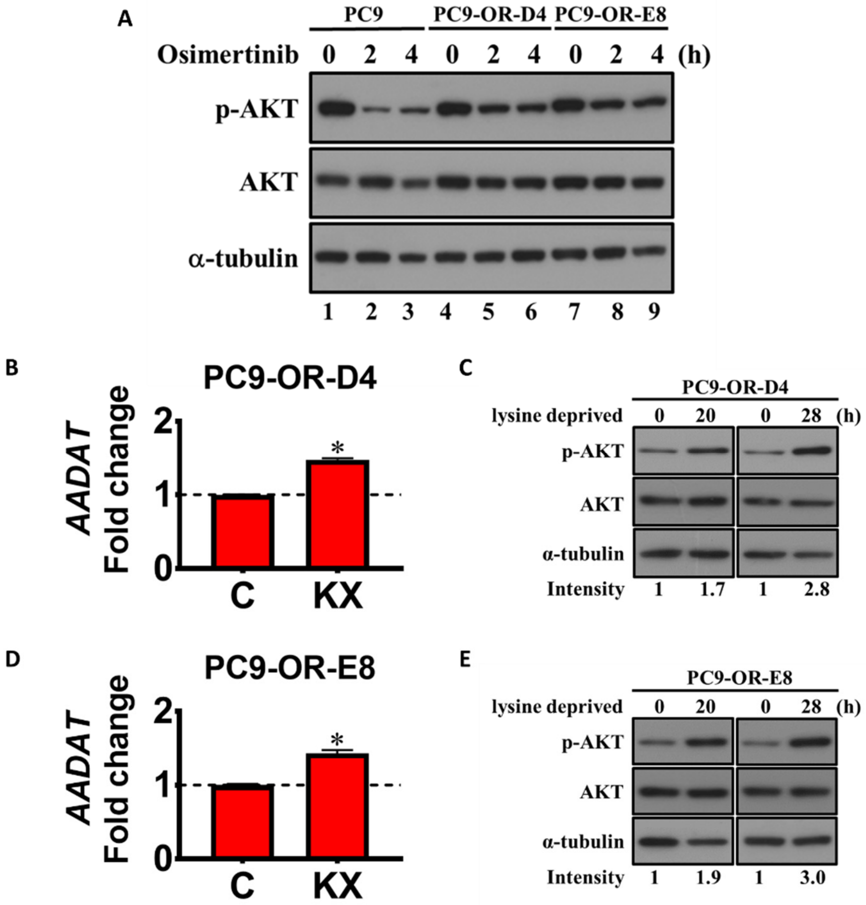

Figure S5. Acquired resistance under osimertinib treatment and the impact of lysine deprivation on *AADAT* and p-AKT/AKT signaling in the PC9 osimertinib-resistant clone. (**A**) PC9 and osimertinib-resistant clones (PC9-OR-D4 and PC9-OR-E8) were treated with osimertinib (25 nM) for 0, 2, and 4 h, and whole-cell extracts were used for the Western blot analysis. The Western blot analysis

was performed using antibodies against phosphor-AKT (p-AKT), AKT, and α-tubulin. (**B**,**D**) PC9-OR-D4 and PC9-OR-E8 were incubated with either the lysine-deprived (0 μM lysine) or control medium (220 μM lysine) for 24 h. The expression of genes involved in lysine catabolism was analyzed using qPCR. The levels of the relative fold change in each lysine-deprived group were normalized to the corresponding control. The results shown are representative of three independent experiments. C, normal RPMI; KX, lysine-deprived RPMI; *AADAT*, kynurenine/α-aminoadipate aminotransferase. The data are presented as the mean ± SEM from three independent experiments. (*, $p < 0.05$ compared with the normal RPMI group). (**C**,**E**) PC9-OR-D4 and PC9-OR-E8 were incubated with lysine-deprived RPMI for 20 and 28 h, and whole-cell extracts were used for the Western blot analysis with antibodies against phosphor-AKT (p-AKT), AKT, and α-tubulin. The Western blot analysis of each relative fold change was measured using the ImageJ software and normalized to each control group. The Western blot analysis was performed using antibodies against phosphor-AKT (p-AKT), AKT, and α-tubulin. Uncropped Western Blots can be found in Figure S8.

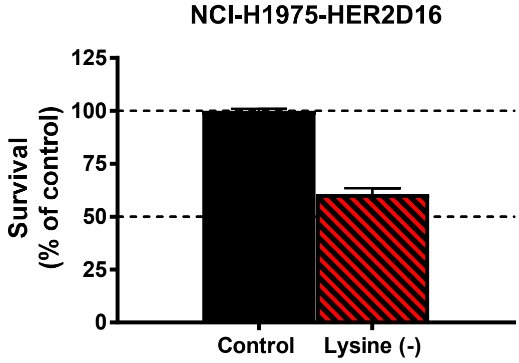

Figure S6. The survival of NCI-H1975-HER2D16 cell lines under lysine deprivation. NCI-H1975-HER2D16 cell lines were incubated with the control medium along with the lysine-deprived medium for 48 h, and the survival was analyzed using SRB assay. The SRB analysis results shown are representative of three independent experiments. SRB, sulforhodamine B.

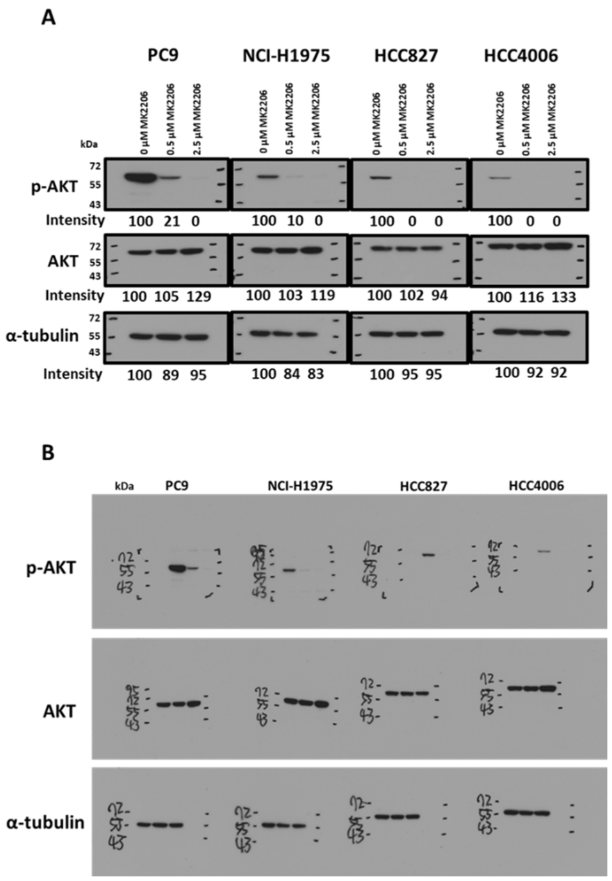

Figure S7. The original Western blot films. **(A)** Figure 5 and **(B)** Figure 6. Lys. Dep., lysine deprivation.

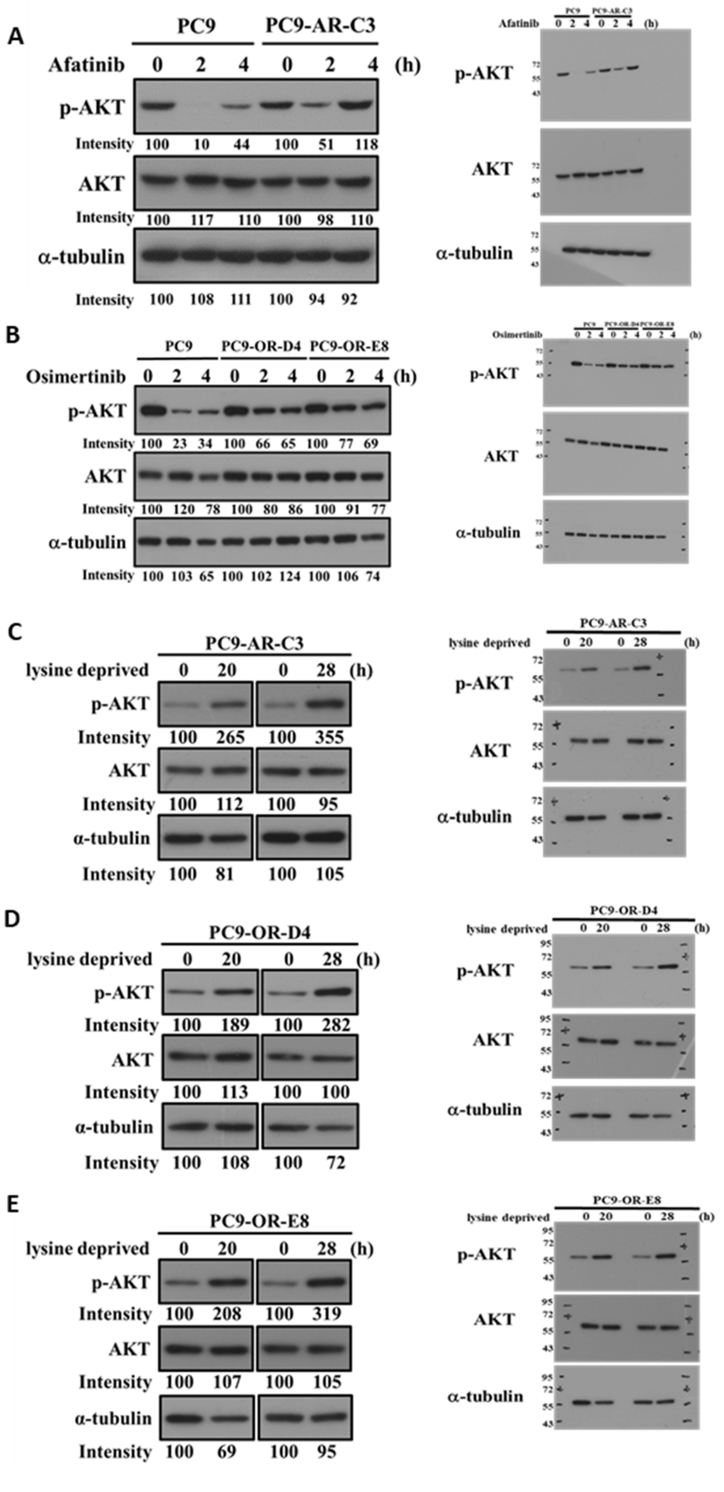

Figure S8. The original Western Blot films. (**A**) Figure S3A. (**B**) Figure S5A. (**C**) Figure S3C. (**D**) Figure S5C and (**E**) Figure S5D.

Publisher's Note: MDPI stays neutral with regard to jurisdictional claims in published maps and institutional affiliations.

© 2020 by the authors. Licensee MDPI, Basel, Switzerland. This article is an open access article distributed under the terms and conditions of the Creative Commons Attribution (CC BY) license (http://creativecommons.org/licenses/by/4.0/).
